# Exploring the Impacts of Living in a “Green” City on Individual BMI: A Study of Lingang New Town in Shanghai, China

**DOI:** 10.3390/ijerph17197105

**Published:** 2020-09-28

**Authors:** Tingting Lu, Matthew Lane, Dan Van der Horst, Xin Liang, Jianing Wu

**Affiliations:** 1 School of International and Public Affairs, Shanghai Jiao Tong University, No.1954 Huashan Road, Shanghai 200030, China; tingting.lu@sjtu.edu.cn (T.L.); wu_jn1288@sjtu.edu.cn (J.W.); 2 China Institute for Urban Governance, Shanghai Jiao Tong University, No.1954 Huashan Road, Shanghai 200030, China; 3 School of Geosciences, The University of Edinburgh, Drummond Street, Edinburgh EH8 9XP, UK; Matthew.Lane@ed.ac.uk (M.L.); Dan.vanderHorst@ed.ac.uk (D.V.d.H.)

**Keywords:** health, sustainable urban planning, BMI, housing tenure choice, urban China

## Abstract

Urban planning and design in the 21st century is increasingly focusing on sustainability, illustrated by the proliferation of greener cities. While operational definitions and the actual planning of these cities can vary considerably (e.g., eco cities and low carbon cities), conceptually, at least, these terms overlap, particularly with regard to how they attempt to achieve both greener infrastructural design and healthier human lifestyles. This paper presents the findings of survey-based research carried out within Lingang New Town in Shanghai in 2019. In the cities of the Global North, the interplay between green infrastructural provision and public health has been of interest, especially in the context of social inequalities; however, there is little research from rapidly urbanizing countries where green urbanism is being increasingly promoted. Using this newly constructed example, we identified a clear positive correlation between moving to a green city and the adoption of healthier lifestyles. The structural equation modelling results suggest that behaviors around the use of green space as well as perceptions of different green space have notable impacts on residents’ physical health, measured by body mass index (BMI). The findings further illustrate systemic inequalities among private housing, rental housing and public housing typologies with regard to the distribution of health benefits.

## 1. Introduction

Urban planning in the 21st century is increasingly focused on sustainability and health [1,2]. This is evidenced by use of terms such as green, eco and low carbon cities across different regions. In principle, these cities are defined by having greener economies, resource consumption, landscapes and communities than traditional cities. While operational definitions and actual configuration can vary considerably, conceptually, at least, these terms overlap with regard to how they attempt to achieve healthier human lifestyles [3,4,5]. The emergence of sustainable urban planning for health can be traced back to the 20th century when Ebenezer Howard planned “garden cities” in the UK and Lewis Mumford promoted “the city beautiful movement” in the US [6,7]. These planning practices depended to an important extent on the provision of safe and accessible green space, as well as people’s opportunities to seek local healthy recreation options. Urban planning’s emphasis on environmental factors has sustained in many regions, with exemplars such as Freiburg in Germany and Hammarby in Sweden, practicing sustainable design for better lives [8,9].

Lately there has been a rise in sustainable urban planning that seeks to invoke “green dreams” against a backdrop of “grey realities” in China [10,11]. This is urgently needed for Chinese cities as societal and economic development is fundamentally constrained by environmental capacities. The sustainable planning practices are particularly seen in the recent construction of holistically designed green cities across the country [12,13]. Existing studies record that over a hundred green cities have been proposed by local governments in China [14,15,16]. Recently, a development scheme of one thousand sustainable communities has been launched by the National Development and Reform Commission [12]. To date, however, few studies have inquired about the social implications of sustainable urban planning in China. One exception is a study of the famous case of Sino-Singapore Tianjin Eco-city, which argued these costly sustainable constructions fail to consider the fate of more marginalized and deprived communities [17]. Beyond this, there is a lack of sufficient focus on the lived experience of residents in newly constructed green cities. The extent to which sustainable urban planning is leading to greener and healthier lifestyles by local inhabitants, remains under-researched. 

A plethora of previous studies found significant relationships between the quality of green space constructed and certain health outcomes, such as obesity, depression, diabetes and cancer [2,18,19,20,21,22,23]. Meanwhile, a considerable plurality and complexity exists in attempting to define what constitutes “quality” green space in terms of the health benefits it can provide in both an environmental (through eco-system services) and a socio-cultural (through recreation and community-building) sense [24,25,26,27,28,29]. The former often measures green space quality by its amounts, planting densities, and gradient vegetation structures, using the satellite image-based normalized difference vegetation index (NDVI) or deep learning-based image segmentation techniques [26,30,31,32]. The latter (socio-cultural) theme analyzes residents’ exposure to green space from both behavioral and attitudinal perspectives [27,31,33,34,35]. In this form, positive exposure to green space nurtures social networking, exercising, leisure, or even commercial and political functions activities. For example, the quality and quantity of amenities in community green space has a negative relationship with obesity prevalence [36]. Secondly, green space users develop subjective perceptions based on the extent their needs are satisfied by the exposure to green space [37]. Encompassing the multi-faceted relationship between green space exposure and urban health outcomes, scholars have argued that a “socio-ecological framework” has emerged as standard practice within the field. In such a framework, the lived experience of green space is identified as one of the most important factors for unlocking health benefits [24]. 

The existing literature has recognized the inequality of health benefit distribution among different social groups in terms of age, race, gender, and income [38,39,40,41,42]. Few studies have explored this systemic inequality in the benefits derived from green space from the perspective of housing modes, however. Previous research in this area has only examined the variation in housing conditions and its impact on health in isolation [36,41,43,44]. In a newly constructed green city, housing mode or housing tenure choice may be associated with health through several potential pathways. First, green cities with sustainable planning methods provide accessible and quality green space as residential advantages to attract new inhabitants. This is similar to the creation of aesthetic suburban new towns that aim to decentralize a city’s central population by the provision of suburban landscapes [45,46]. The positive exposure to green space may encourage residents to purchase or rent housing in the green city. Second, different housing modes are associated with various greening intervention in terms of green space proximity, green space types and green space quality. Prior studies have shown that public housing units often have little or no vegetation while private housing in gated communities guarantees access to exclusive and amplified green space [36,47,48]. Consequently, public housing tenants and private housing owners develop different exposure to and perception of green space. The variation in housing mode nurtures distinct lifestyles in terms of exercising and leisure, leading to different public health outcomes. For example, a public housing tenant may suffer from problems of obesity [36]. Conversely, a homeowner of a gated community may enjoy high-end green provisions and thus develop a positive sense of wellbeing [49]. Housing mode thus may serve as a mediation variable between one’s green lifestyle and their level of health.

### Case Study

This paper aims to examine residents’ health benefits from moving to a brand-new green city—Lingang New Town (LNT)—in Shanghai, thereby providing existing discussions on the relationship between sustainable urban planning and health with a new perspective rooted in housing modes. LNT is one of the most strategically important mega-urban projects in China [50,51,52]. It is located 30 km southeast of central Shanghai, on land reclaimed from the sea, covering an area of 315 km^2^. Historically, it was a peripheral farming area, and developed modern fish farming to feed Shanghai during its rapid industrialization. As part of suburbanization, LNT framed its agenda as a new “garden city” in 2002, emphasizing an environmental message as part of this narrative [53]. Five functional zones have been planned, with a central residential zone and a heavy-equipment manufacturing zone providing a modern environment while the rest of the zones remain as traditional townships. The original planning of LNT aimed to attract 0.8 million new residents by the end of 2020 [52]. In 2010, LNT was upgraded to a low carbon plot city by the Shanghai Municipal Government [54]. Many pivotal green infrastructural constructions began to unfold at this stage. Among them, 8.6 km of greenways for walking and cycling, as well as the artificial Dishui Lake park of 5.6 km^2^, and an affiliated 20 km^2^ of green space in the central residential zone, were presented as strategically important green infrastructure. Quickly, high-quality green space extended into different functional zones, covering over 30% of the land use, turning LNT into a much greener area than much of metropolitan Shanghai. Recently, Shanghai Municipal Government consulted a UK-based design company (Arup) and local planning institutions to reinforce LNT as a landmark of global green development, with ambitions for green space and waterfront space to cover 65% of its land area [55]. Meanwhile, the administrative committee of LNT has endeavored to advertise a green lifestyle and culture to the public, for which better health outcomes for residents act as a clear incentive [54].

**Figure 1:**
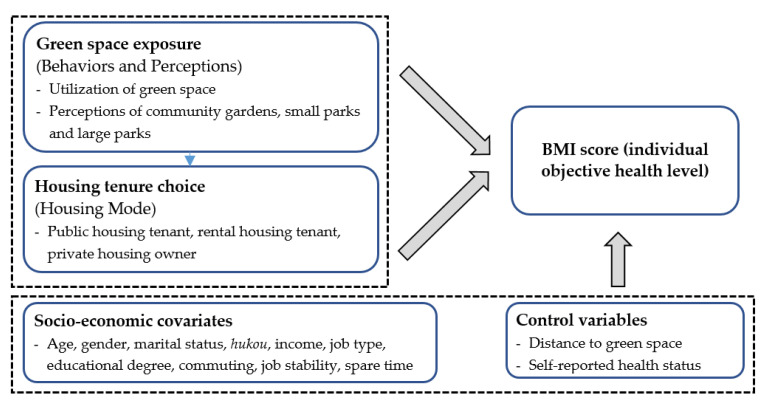
Theoretical framework.

The case study of LNT offers unique access to the physical health and green space perspectives of residents who have taken the decision to move to a new urban area explicitly targeting sustainable urban planning outcomes. Our intellectual contribution is therefore twofold. Firstly, we take a nuanced and attentive approach to the socio-cultural dimension of green cities, exploring the perception, behavior, and health of people who come to reside in these green spaces. Secondly, from an urban planning perspective, we elevate the importance of indicators of health outcomes in assessing the value-added of urban green space planning and delivery in brand new green cities. Using body mass index (BMI) as an indicator, we hypothesize that an individual’s health level is affected by the experience of green space, and such a relationship is mediated by one’s housing tenure choice. As shown in Figure 1, this paper examines both the direct effects of green space exposure and the indirect effects mediated by housing mode on BMI by using the structural equation modelling method. 

## 2. Materials and Methods 

### 2.1. Data

**Figure 2:**
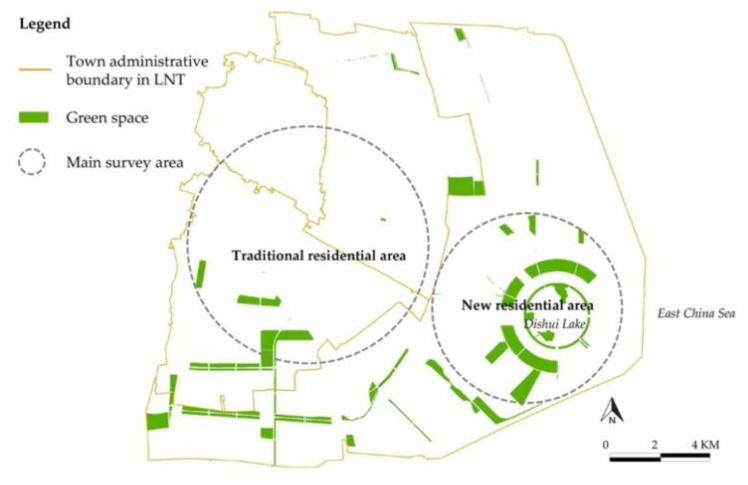
Main green space distribution in the survey area in Lingang New Town (LNT).

The data for this paper were collected by a household questionnaire survey conducted in LNT from March to May 2019. A stratified sampling method was applied because LNT planned two residential areas, namely a new residential area and a traditional residential area (see Figure 2). The former was near the Dishui Lake, mostly in the form of high-rise buildings that were affiliated with community gardens and accessible parks. In contrast, the latter residential area consisted of smaller and older neighborhoods in three towns, with less provision for green spaces of all kinds.

The new residential area surveyed included eight neighborhoods with a total of 17,494 registered households. We selected three out of the eight neighborhoods with the highest occupation rate and distributed 100 questionnaires to each as an indoor household survey. Questionnaires were submitted for completion by new inhabitants, either heads of households or their spouses. The traditional residential area surveyed had 42 neighborhoods with a total of 16,877 registered households. We distributed 150 questionnaires in four public spaces, aiming to seek new inhabitants of LNT in the wide range of neighborhoods in this residential area. All questionnaires were completed onsite. In total, we achieved 427 full responses of which 403 were deemed valid after missing value processing.

### 2.2. Measures

The questionnaire included questions relating to residents’ socio-demographic status, housing profiles, health and lifestyle. Specifically, the following set of variables was measured:

(1) Outcome variable: individual BMI was used as the outcome variable. It is one of the most important proxy indicators used to represent one’s physical health level in many relevant studies [23,29,56]. Respondents were asked to report their height and weight for measuring BMI as a continuous variable.
(2) Variables of (green) lifestyle: both behaviors and perceptions related to the exposure of green space in LNT were measured in this research to reflect people’s green lifestyles. The behavior aspect was measured by querying residents’ frequency of using green space before moving to LNT and their current use of green space in LNT, with four-scale answers provided (1 = “less than once a week”, 2 = “once or twice a week”, 3 = “three to six times a week”, 4 = “everyday”). We inspected residents’ perceptions of green space in LNT in seven dimensions that were acknowledged as green space’s key functions in the sustainable urban planning code: exercising, safety, accessibility, social interaction, commerce, public events, and environment quality. Specifically, residents were asked four sets of questions, including to what extent the specific dimension of green space is important to them, and to what extent they are satisfied with every dimensional function of green space of a specific kind. Three kinds of green space were inspected, respectively, namely community gardens (in community), small parks (nearby community), and large parks, covering most of the green infrastructure types in LNT. Answers on a Likert scale were provided for these 28 questions, with the score ranging from 1 (indicating extremely unimportant/unsatisfied) to 7 (indicating extremely important/satisfied).
(3) Housing mode variable: residents’ housing tenure choice in LNT was measured to constitute the housing mode variable. Overall, there were three housing modes identified, namely private housing, rental housing and public housing. The private housing mode referred to residents who owned a local private property. The rental housing mode referred to tenants who rented from the housing market. Public housing mode represented tenants who obtained subsidized housing provided by the local government. This housing mode was mostly provided to employees of state-owned enterprises in LNT as temporary accommodation.
(4) Covariates: respondents’ individual profiles were considered as confounding variables. In terms of socio-demographic status, we surveyed the heads of household or their spouses for age, gender, marital status, *hukou* status, educational level and the household monthly income level. It is important to note that *hukou* status is one of the most crucial indicators of individual socio-economic capabilities. *Hukou* is a household registration system in China that defines one’s right to different socio-economic benefits. For example, the *hukou* origin determines the access to local socio-economic welfare support, such as education allowances and medical care. Furthermore, only non-agricultural *hukou* holders can have urban welfare support, which is of a much higher standard than rural forms. In this research, we defined a respondent as a migrant if his/her *hukou* origin was outside Shanghai. The type of *hukou* was categorized into agricultural and non-agricultural based on the type of *hukou* registration. A respondent with a college or above degree was regarded as having a high educational level. Household monthly income in Chinese currency “RMB” was classified into six levels (1 = “less than 1000“, 2 = “1000–4999”, 3 = “5000–10,000”, 4 = “10,001–20,000”, 5 = “20,001–30,000”, 6 = “more than 30,000”). As for factors of work and life, we measured respondents’ job type, commuting time, amount of spare time and length of time spent living in LNT. Specifically, working for the public sector was considered as a stable job type in Chinese cities. One’s amount of spare time was likely to be affected by his/her employment status and commuting time. The residence length in LNT helped to verify respondents as new inhabitants of LNT.
(5) Control variable: a few variables relating to the lived experience in LNT were considered as control variables for analyzing BMI. First, respondents were asked to report their subjective perception of individual health, ranging from “completely unhealthy”, “relatively unhealthy”, “relatively healthy”, to “completely healthy”. The walking time from home to the nearest green space was surveyed, with four answers provided (1 = “less than 10 min”, 2 = “11–20 min”, 3 = “21–30 min”, 4 = “more than 30 min”).

### 2.3. Model Construction

Structural equation modelling was used to estimate the effect of green lifestyles on one’s BMI with housing mode acting as a mediation variable. Individual socio-economic status was considered as covariates influencing housing mode and BMI, respectively. Given that BMI can also be affected by the location of green space and subjective health level, both the walking time to nearest green space and the self-reported health level were added as control variables. 

## 3. Results

### 3.1. Descriptive Statistics

Table 1 shows residents’ socio-economic profiles, exposure to green space and health level by housing mode. Private, rental and public housing modes, respectively, occupy 56.8%, 29.3%, and 13.9% of the surveyed households. The private housing mode reports the highest proportions for variables of married (84.7%) and non-agricultural *hukou* (86.0%) as well as a relatively senior average age, compared to the rest. The rental housing mode provides accommodation for the largest proportions of the migrants (77.1%), the agricultural *hukou* holders (43.2%), and households of relatively lower income as compared to other housing modes. The public housing mode concentrates the highly educated (89.3%), and high-income earners, and the single and young as compared to the rest. In terms of work and life, the private housing mode has the highest proportion of full-time workers (88.2%) and the longest work-home travel duration. Many residents of the private housing mode may have less spare time than their counterparts of other housing modes. With regard to the green lifestyle variables, residents of the public housing mode have the highest frequency of using green space both before moving to LNT and currently, despite having the longest travel time to the nearest green space. Residents of the rental housing mode report a higher level than others for self-evaluated health and the experience of green space in LNT.

With regard to local health, it is identified that the average BMI score of the sampled 403 respondents is 22.6, ranging from 15.6 to 32.9. According to the BMI standards announced by the Chinese Ministry of Health, low weight, normal weight, overweight, and obesity are, respectively, considered as BMI < 18.5, 24 > BMI ≥ 18.5, 28 > BMI ≥ 24 and BMI ≥ 28 [57]. The results of our survey show that 66.0% of the respondents have a healthy weight, while 6.0% of the respondents are of a low weight, 24.5% are overweight and 3.5% are obese. According to the National Health and Family Planning Commission, the adult overweight rate was around 30.1% and the obesity rate was about 11.9% in 2012 in China [56]. It seems that respondents in LNT demonstrate a relatively positive level of health in terms of BMI, as the rates of overweight and obese individuals are 5.6% and 4.8% less than the national average. 

### 3.2. Analysis on Behaviors and Perceptions of Green Space Exposure

Table 2 demonstrates that moving to LNT is improving residents’ behavior regarding green space use. This is indicated by the relative change of frequency in using green space before and after moving to LNT. The majority (57.3%) of respondents barely used green space before (i.e., once every several weeks), but this group has now dropped to a minority of 39.0%. That means that about a third of these previously inactive residents (in terms of green space) have changed their behavior and are now weekly green space users. Those who reported using green space multiple times a week almost doubled, with “1–2 times per week use” rising by 48.1% and “3–6 times per week use” rising by 46.3%. The small group of residents who reportedly used green space every day did not change much, increasing only by 14.5%. If we assume that increased green space use is a proxy indicator for a greener lifestyle, then we can posit that the move to LNT was particularly beneficial to those who were relatively inactive. 

Furthermore, principle component analysis was employed to reduce the dimensionality of residents’ perceptions of green space in LNT. Table 3 demonstrates five components after applying a varimax rotation. They account for 67.5% of the cumulative variance of 28 attributes. The Cronbach’s alpha score, and the Kaiser–Meyer–Olkin score, respectively, reach 0.933 and 0.894, showing a high internal consistency of attributes and an effective dimensional reduction effect by the analysis. Specifically, Component I has high positive loadings on attributes of exercising, safety and environmental quality of community gardens, indicating a group of residents who are satisfied by the exclusive green space provided within communities because of these functions. Component II has high positive loadings on safety and accessibility attributes of small parks located near to communities. In addition to safety and accessibility, Component III reports the attribute of quality environment of large parks as high loadings. The concentration of high loadings is found in the attributes of commerce and public events in green space for Component IV, while clustering at other dimensions of value for Component VI. Two components show contradictory perceptions of green space, that is, while Component VI reflects social values provided by green space, Component V appreciates the physical values of green space in general. 

### 3.3. Structural Equation Model Results

Table 4 reflects the results of the structural equation modelling. Model 1 shows the direct effect of green lifestyles on the private housing mode and BMI. Given that green space perception may have indirect effect on BMI through housing modes, we added the private housing mode as a mediation variable in model 2. We replaced the housing mode with public housing in model 3 for a robustness test. Model fit statistics of the three models reveal a good fit of structural equation modelling. The root mean square error of approximation, respectively, reached 0.060, 0.024, and 0.075, with all three comparative fixed indexes being larger than 0.95.

Variables relating to green space exposure associate differently to the private housing mode in model 2 and to the public housing mode in model 3. Specifically, residents’ relative change in frequency of using green space after moving to LNT shows a positive correlation with the private housing mode (β = 0.060, S.E. = 0.028, *p* = 0.035), but has a negative non-significant effect on the public housing mode after controlling for socioeconomic variables. As for the effects of green space exposure on BMI, perceptions of green space are significantly correlated with BMI in three models. For example in model 2, perception of large parks positively correlates to BMI (β = 0.169, S.E. = 0.087, *p* = 0.053), while the perception of small parks has a negative association with BMI (β = −0.174, S.E. = 0.100, *p* = 0.082).

With regard to the socio-economic influences on BMI in model 1, females and the highly educated have a significantly lower level of BMI, with the coefficient of gender being −2.092 (*p* < 0.01) and the coefficient of educational attainment being −1.070 (*p* < 0.01), compared to the male groups and respondents without a college degree. A one-year increase in age increases individual BMI by 0.054 standard deviation (*p* < 0.01). Socio-economic differentiation is clearly seen in the regressions of the housing mode. The private housing mode is attracting the married (β = 0.162, S.E. = 0.060, *p* = 0.007) and the non-agricultural *hukou* holders (β = 0.107, S.E. = 0.060, *p* = 0.073), while public housing is significantly associated with migrants (β = 0.090, S.E. = 0.039, *p* = 0.020) and the public sector (β = 0.080, S.E. = 0.038, *p* = 0.037). 

More importantly, choosing a private housing mode is found to have a direct and positive effect on respondents’ BMI (β = 0.617, S.E. = 0.279, *p* = 0.027), even with the time to the nearest green space and the level of self-evaluated health held constant. Model 3 reports the results with the public housing mode replacing the private housing mode. In contrast to private housing, the public housing mode negatively affects individual BMI (β = −0.947, S.E. = 0.367, *p* = 0.009). Relations between the experience of green lifestyles and individual BMI are not altered when the housing mode is replaced, thus demonstrating the robustness of the model results.

## 4. Discussion

There are three main findings regarding the health outcomes of sustainable urban planning in this study. First, we find evidence that more green space nurtures healthier lifestyles. This is illustrated by the fact that a large proportion of previously inactive groups (e.g., used green space once a few weeks) have been encouraged to use green space at a higher frequency than previously. The adoption of green lifestyle choices from green space exposure is likely to be stimulated by effective sustainable urban planning for green infrastructure, as the prior literature suggests [58]. In the case of LNT, green space has been made accessible near residences for the majority of the new population, as this survey revealed that over 80% of residents were able to access green space within 20 min by walking. To a certain extent, LNT has been transformed from an industrial land at the urban periphery to a green new town with healthier lifestyles through the ample provision of green infrastructure. 

**Figure 3:**
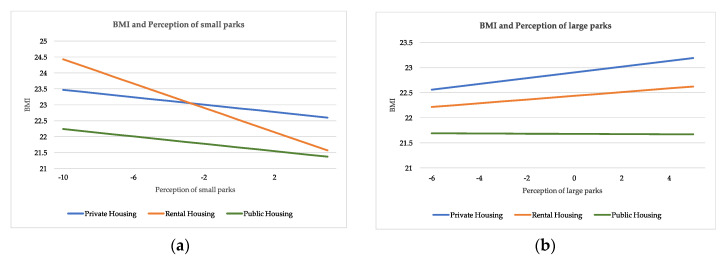
(**a**) Predicted relationship between individual body mass index (BMI) and the perception of small parks; (**b**) predicted relationship between individual BMI and the perception of large parks.

Second, sustainable urban planning generates varied perceptions regarding green space exposure. The provision of safe, accessible, and high-quality green space appears to meet residents’ demands. Importantly, it is the effective exposure to small parks that mitigate against the risks of becoming (or staying) overweight and obese. In LNT, the planning and wide distribution of small parks in residential neighborhoods incentivizes many local users on a regular basis. In contrast, large parks may have opposite outcomes because they are of a limited amount and have unequal accessibility to all users. Figure 3 displays the predicted negative relationship between small park perception and BMI, the positive relationship between large park perception and BMI, as well as the differences among three housing modes.

Third, residents’ BMI differs among public housing, private housing, and rental housing. This is likely influenced by different lifestyles that relate to a housing tenure choice. Private homeowners have a relatively high BMI. They are most likely to be stable and occupied with work, leaving little spare time to experience green space. It is the private housing mode that has the highest proportion of senior and well-established families. This is consistent with national level statistics in China in that obesity and diabetes become more common as residents become older [56]. Many rental housing tenants have a lower BMI than owners. This is possibly because tenants are mostly new to LNT, and have a relatively high frequency of using green space because of this (self-identified) excellence in green space provision. Especially in public housing, tenants tend to have a low level of BMI. This differs from studies based in a Western context in which tenants of public housing can suffer from being overweight [36,47]. In urban China, green cities are state-led developments, which usually provide government-subsidized housing for accommodating public sector staff and attracting talented workers. In fact, in LNT, many residents in public housing are young and highly educated in the field of low-carbon technological development. However, it is important to note that the unhealthy status of low weight is mostly found in migrants—occupying 66.7%—who live in rental housing. Being a migrant means having no access to welfare provided for local citizens, e.g., healthcare benefits. Discrimination against migrants in health benefit distribution can still be seen in this form of sustainable urban planning. 

## 5. Conclusions

While green cities are expected to play dual roles as economic growth engines while implementing sustainable development in practice, there are increasing concerns about the social outcomes of this global trend within urban planning. Particularly, questions around green infrastructure’s role as a catalyst for healthy lifestyles through fair and equal access to quality green infrastructure need to be asked [24,59]. In urban China, the emerging development of green cities has emphasized this infrastructural approach to positive, sustainable outcomes [11,12,16]. The pursuit of “green dreams” by motivated residents has been met with considerable investment in sustainable planning and design strategies. Illustrating how the design-based provision of green space has encouraged more frequent use by residents after they move in, this study emphasizes the value of not only incorporating green space into the promotion of green lifestyles, but doing so in such a way as to stress how residents experience it in both physical and socio-cultural ways.

Our analysis yields two conclusions regarding how sustainable urban planning relates to residents’ health. First, green city construction is contributing to an objective health level for new residents, represented by relatively high proportion of normal BMI. Specifically, BMI is clearly linked to one’s socio-economic profile and to green space-related mindsets and behaviors, especially those related to exercising, a sense of safety, and the environmental quality of small parks. Second, there is a trend in socio-spatial inequality in terms of the distribution of health benefits. While private homeowners are more likely to have elevated BMI scores, disadvantaged social groups, such as low-skilled migrants, have below normal BMI scores. The unevenness of one’s lifestyle and health can be exacerbated by local planning. To avoid marginalizing disadvantaged social groups [17,52], green city planning and design should focus on achieving a good distribution of green space access throughout green city neighborhoods, prioritizing this over the construction of larger spaces with prescribed uses for manifestation. A focus should be placed on presenting green space as a “blank canvas”, allowing residents to invent a sense of place which harmonizes the physical and socio-cultural benefits of green space access. 

Finally, it is important to couch our findings within the context of the current COVID-19 pandemic (as an acute representation of a systemic challenge to urban public health) and the implications this has for both enforced and self-managed behavior changes in how green space is accessed and utilized. In addition to the relationships with green space access and use identified in this study, socio-economic status and BMI have also been shown to indicate various vulnerabilities to the COVID-19 risk levels. The need for increased social distancing levels when utilizing green space in order to limit flu-like virus transmission will therefore unevenly impact certain cohorts of the urban population. With an increasing emphasis on the need to “build back better” following the pandemic, new urban developments (anywhere in the world, not just in China) need to pay extra attention to green space provision. How this green space is designed for and utilized by different segments of the urban population will remain an important area for furthering empirical analysis and conceptual understanding.

## Figures and Tables

**Table 1:** Summary statistics (by housing tenure choice).

	Description	Range	Housing Tenure Choice			F Value
			Private (*n* = 229)	Rental (*n* = 118)	Public (*n* = 56)	
Age	Mean (S.D.)	18–80	36.4 (9.0)	34.3 (11.0)	32.8 (10.0)	3.9 *
Gender	Female (%)		57.6	42.4	44.6	
Marital status	Married (%)		84.7	65.3	55.4	
*Hukou* origin	Non-Shanghai (%)		32.3	77.1	64.3	
*Hukou* type	Non-agriculture (%)		86.0	56.8	69.6	
Educational degree	College or above (%)		84.7	71.2	89.3	
Job type	Public sector (%)		53.3	28.0	57.1	
	Other sector (%)		46.7	72.0	42.9	
Employment status	Retired (%)		3.1	1.7	3.6	
	Part-time (%)		8.7	17.0	8.9	
	Full-time (%)		88.2	81.3	87.5	
Commuting minutes	Mean(S.D.)	2–120	26.1 (19.8)	20.8 (16.6)	18.7 (10.3)	5.9 **
Household monthly income level	Mean (S.D.)	1–6	4.1 (1.1)	3.7 (1.2)	4.1 (0.9)	4.1 *
Year living in LNT	Mean (S.D.)	0–18	4.2 (3.2)	2.2 (1.6)	1.6 (1.1)	35.8 ***
Frequency of using green space before	Mean (S.D.)	1–4	1.6 (0.9)	1.7 (0.9)	1.8 (0.9)	1.3
Frequency of using green space now	Mean (S.D.)	1–4	1.9 (0.9)	1.9 (0.9)	2.0 (1.0)	0.22
Distance to nearest green space	Mean (S.D.)	1–4	1.7 (0.9)	1.8 (1.0)	2.1 (1.1)	4.6*
Self-reported health level	Mean (S.D.)	1–4	2.9 (0.7)	3.1 (0.8)	3.0 (0.9)	1.6
BMI	Mean (S.D.)	15.6–32.9	22.9 (2.9)	22.5 (2.8)	21.7 (2.4)	4.4*

Note: * *p* < 0.05, ** *p* < 0.01, *** *p* < 0.001.

**Table 2:** Relative change of green space use, before and after residents moving to LNT.

Frequency of Using Green Space	Before Moving to LNT (%)	After Moved to LNT (%)	Relative Change (%) ^1^
Everyday	6.2	7.1	+14.5
3–6 times per week	9.5	13.9	+46.3
1–2 times per week	27.0	40.0	+48.1
Once in a few weeks	57.3	39.0	−31.9

^1^ relative change is measured by [(after–before)/before]*100%.

**Table 3:** Principal components of green space perceptions.

Component	Variance	Loaded Items (>0.40)	Percentage of Explained Variance	Generative Content
I	4.842	Exercising (0.402), safety (0.413), quality environment (0.430)	0.153	Perception of community garden
II	4.778	Safety (0.486), accessibility (0.459)	0.143	Perception of small parks
III	4.460	Safety (0.457), accessibility (0.421), quality environment (0.413)	0.137	Perception of large parks
IV	4.052	Commerce (0.421), public events (0.425)	0.133	Social values of green space in general
V	3.137	Safety (0.480), accessibility (0.449), quality environment (0.436)	0.109	Physical value of green space in general

**Table 4:** Results of structural equation modelling.

	Model 1		Model 2		Model 3	
	Private Housing	BMI	Private Housing	BMI	Public Housing	BMI
	Coef. (S.E.)	Coef. (S.E.)	Coef. (S.E.)	Coef. (S.E.)	Coef. (S.E.)	Coef. (S.E.)
**Housing tenure choice**						
Private housing				0.617 ** (0.279)		
Public housing						−0.947 ** (0.367)
Green lifestyles						
Relative change of using green space	0.060 ** (0.028)	−0.116 (0.160)	0.060 ** (0.028)	−0.152 (0.160)	−0.021 (0.022)	−0.134 (0.159)
Frequency of using green space now	−0.026 (0.031)	0.230 (0.179)	−0.026 (0.031)	0.248 (0.178)	0.023 (0.023)	0.262 (0.178)
Perception of community garden	−0.009 (0.015)	0.042 (0.086)	−0.009 (0.015)	0.048 (0.085)	0.015 (0.012)	0.057 (0.085)
Perception of small parks	−0.020 (0.018)	−0.176 * (0.100)	−0.002 (0.018)	−0.174 * (0.100)	−0.016 (0.014)	−0.189 * (0.100)
Perception of large parks	−0.007 (0.016)	0.164 * (0.088)	−0.007 (0.016)	0.169 * (0.087)	−0.004 (0.012)	0.161 * (0.087)
Social values of green space in general	0.001 (0.014)	−0.109 (0.079)	0.001 (0.014)	−0.111 (0.078)	−0.001 (0.046)	−0.111 (0.078)
Physical value of green space in general	0.008 (0.013)	−0.002 (0.072)	0.008 (0.013)	−0.008 (0.072)	0.006 (0.010)	0.001 (0.072)
**Covariates**						
Age	−4.19 × 10^−4^ (0.003)	0.054 *** (0.018)	−4.19 × 10^−4^ (0.003)	0.055 *** (0.018)	−0.001 (0.002)	0.053 *** (0.018)
Educational level (college and above = 1)	−0.034 (0.066)	−1.070 *** (0.372)	−0.034 (0.066)	−1.056 *** (0.370)	0.068 (0.050)	−1.017 *** (0.370)
Gender (female = 1)	0.085 * (0.046)	−2.092 *** (0.259)	0.085 * (0.046)	−2.143 *** (0.259)	−0.032 (0.035)	−2.122 *** (0.258)
*Hukou* origin (migrant = 1)	−0.316 *** (0.051)	−0.584 ** (0.285)	−0.316 *** (0.051)	−0.392 (0.296)	0.090 ** (0.039)	−0.503 * (0.285)
*Hukou* type (non-agriculture = 1)	0.107 * (0.060)	0.395 (0.336)	0.107 * (0.060)	0.332 (0.335)	−0.011 (0.046)	0.391 (0.333)
Marital status (married = 1)	0.162 *** (0.060)	0.454 (0.340)	0.162 *** (0.060)	0.353 (0.341)	−0.123 *** (0.046)	0.341 (0.340)
Level of household monthly income	0.014 (0.021)	0.140 (0.119)	0.014 (0.021)	0.136 (0.118)	0.014 (0.016)	0.160 (0.118)
Job type (public sector = 1)	−0.004 (0.050)	−0.220 (0.282)	−0.004 (0.050)	−0.218 (0.280)	0.080 ** (0.038)	−0.148 (0.281)
Commuting time	0.002 *** (0.001)	0.005 (0.005)	0.002 *** (0.001)	0.004 (0.005)	−0.001 (0.001)	0.004 (0.005)
Employment status (retired = 1)	0.018 (0.163)	−1.329 (0.917)	0.018 (0.163)	−1.347 (0.912)	0.114 (0.124)	−1.234 (0.911)
**Control variables**						
Self-reported health		−0.449 *** (0.162)		−0.443 *** (0.161)		−0.459 *** (0.161)
Time to nearest green space		0.210 (0.133)		0.232 * (0.133)		0.253 * (0.133)
**Constant**	0.435 (0.149)	22.262 (1.020)	0.435 *** (0.149)	21.919 *** (1.026)	0.096 (0.114)	22.273 *** (1.012)
**RMSEA**	0.060		0.024		0.075	
**CFI**	0.979		0.998		0.968	

Note: * *p* < 0.1, ** *p* < 0.05, *** *p* < 0.01.
